# Cell-Free Expression of *De Novo* Designed Peptides That Form β-Barrel Nanopores

**DOI:** 10.1021/acsnano.2c07970

**Published:** 2023-02-02

**Authors:** Shoko Fujita, Izuru Kawamura, Ryuji Kawano

**Affiliations:** † Department of Biotechnology and Life Science, Tokyo University of Agriculture and Technology, Tokyo184-8588, Japan; ‡ Graduate School of Engineering Science, Yokohama National University, Yokohama240-8501, Japan

**Keywords:** nanopore, cell-free synthesis, lipid bilayer, peptide sensing, molecular dynamics simulations

## Abstract

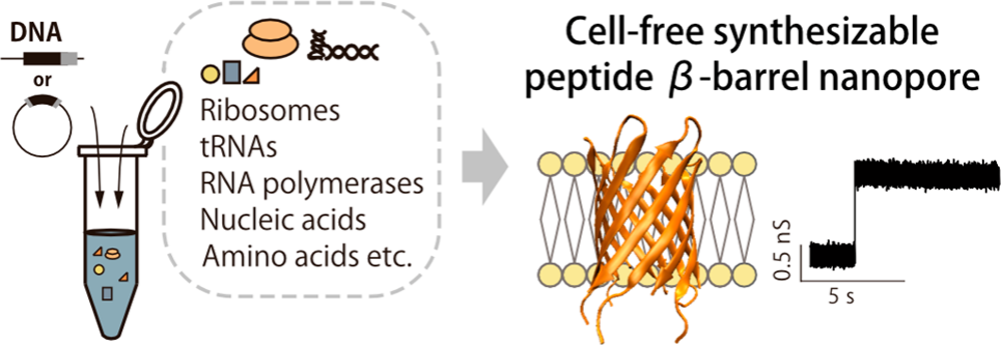

Nanopore sensing has attracted much attention as a rapid, simple, and label-free single-molecule detection technology. To apply nanopore sensing to extensive targets including polypeptides, nanopores are required to have a size and structure suitable for the target. We recently designed a *de novo* β-barrel peptide nanopore (SVG28) that constructs a stable and monodispersely sized nanopore. To develop the sizes and functionality of peptide nanopores, systematic exploration is required. Here we attempt to use a cell-free synthesis system that can readily express peptides using transcription and translation. Hydrophilic variants of SVG28 were designed and expressed by the PURE system. The peptides form a monodispersely sized nanopore, with a diameter 1.1 or 1.5 nm smaller than that of SVG28. Such cell-free synthesizable peptide nanopores have the potential to enable the systematic custom design of nanopores and comprehensive sequence screening of nanopore-forming peptides.

Pore-forming proteins reconstituted in a bilayer lipid membrane (BLM) offer characteristic information on molecules passing electrophoretically through the pore, via the level of the blocking current. There has been considerable attention given to this nanopore sensing technology because of its potential as a powerful tool for label-free single-molecule detection.^1−5^ The α-hemolysin (αHL) nanopore, which is derived from *Staphylococcus aureus* and is one of the most commonly used nanopore proteins to date, has been applied in the single-molecule recognition of nucleotides,^6−10^ peptides,^11−14^ proteins,^15,16^ etc.^17,18^*Mycobacterium smegmatis* porin A (MspA) was reported as nearly ideal for the detection of single-stranded nucleic acid strands^19^ and has been investigated in the realization of a nanopore sequencer. Based on these studies, the company Oxford Nanopore Technology has recently commercialized a nanopore DNA sequencer using a CsgG nanopore instead of the MspA nanopores.^20^ Having achieved DNA sequencing using nanopore technology, the next target is the sequencing of proteins that have amino acids with fewer structural differences than DNA.^21−23^ Several attempts toward peptide and protein sequencing using aerolysin,^24^ FraC,^25^ and MspA^26,27^ nanopores have recently been reported.

The selectivity of nanopore sensing is mainly dependent on the pore configuration and the interaction between the pore surface and the target molecules. Although several pore-forming proteins in nature have been investigated for polypeptide detection,^28,29^ challenges remain. The bottom-up design of nanopore-forming materials is a promising method to enable custom-made nanopores for a given target molecule, leading to recent reports on the *de novo* design of nanopore-forming proteins/peptides. For example, a *de novo* α-helical barreled peptide ion channel and nanopore have been reported.^30−32^ Baker and co-workers have reported a β-barrel protein with eight strands, engineered using computational sequence design with several strategies and optimization of the loop sequence.^33^

We previously reported a *de novo* designed β-hairpin peptide (SVG28) that assembles to form a β-barrel structure in the BLM with a diameter of predominantly 1.7 nm, and with the ability to discriminate poly-l-lysine of different chain lengths.^34^ To functionalize and improve the resolution of the detection signals for future polypeptide detection, we would like to investigate systematic design based on SVG28 sequences and to evaluate their capabilities. The SVG28 has 28 residues and is synthesized by solid-phase synthesis. The high hydrophobicity and aggregation propensity of SVG28 make it difficult to purify using typical methods; therefore, the isoacyl dipeptides method^35^ was needed to synthesize SVG28. The complex and time-consuming procedures required for SVG28 synthesis is a major challenge in the systematic sequence optimization, so herein we attempted to use the cell-free protein synthesis system, which enables the synthesis of proteins and peptides *in vitro* using transcription–translation.

Since around 1960, arbitrary polypeptides have been synthesized by adding mRNA or nucleic acid fractions to cell lysate.^36−38^ However, peptides do not form folded structures and tend to be degraded by proteases contained in the lysate,^39^ and therefore are not suitable in this cell-free synthesis. Shimizu and co-workers have reported a reconstituted cell-free synthesis system (PURE system) using the individually purified proteins that contribute to transcription and translation, resulting in a higher yield than cell lysate.^40^ This system does not contain protease and can also synthesize peptides.

In this study, we introduced hydrophilic substitutions to SVG28 and expressed the designed peptides using the PURE system. The pore-forming ability, pore size, and noise of hydrophilic variants were analyzed using electrophysiology to evaluate the effect of the hydrophilic mutations. The applicability of the hydrophilic nanopores to protein sequencing was also verified by single molecule detection of oligopeptides.

## Results and Discussion

### Design of Hydrophilic Variants of SVG28

**Figure 1:**
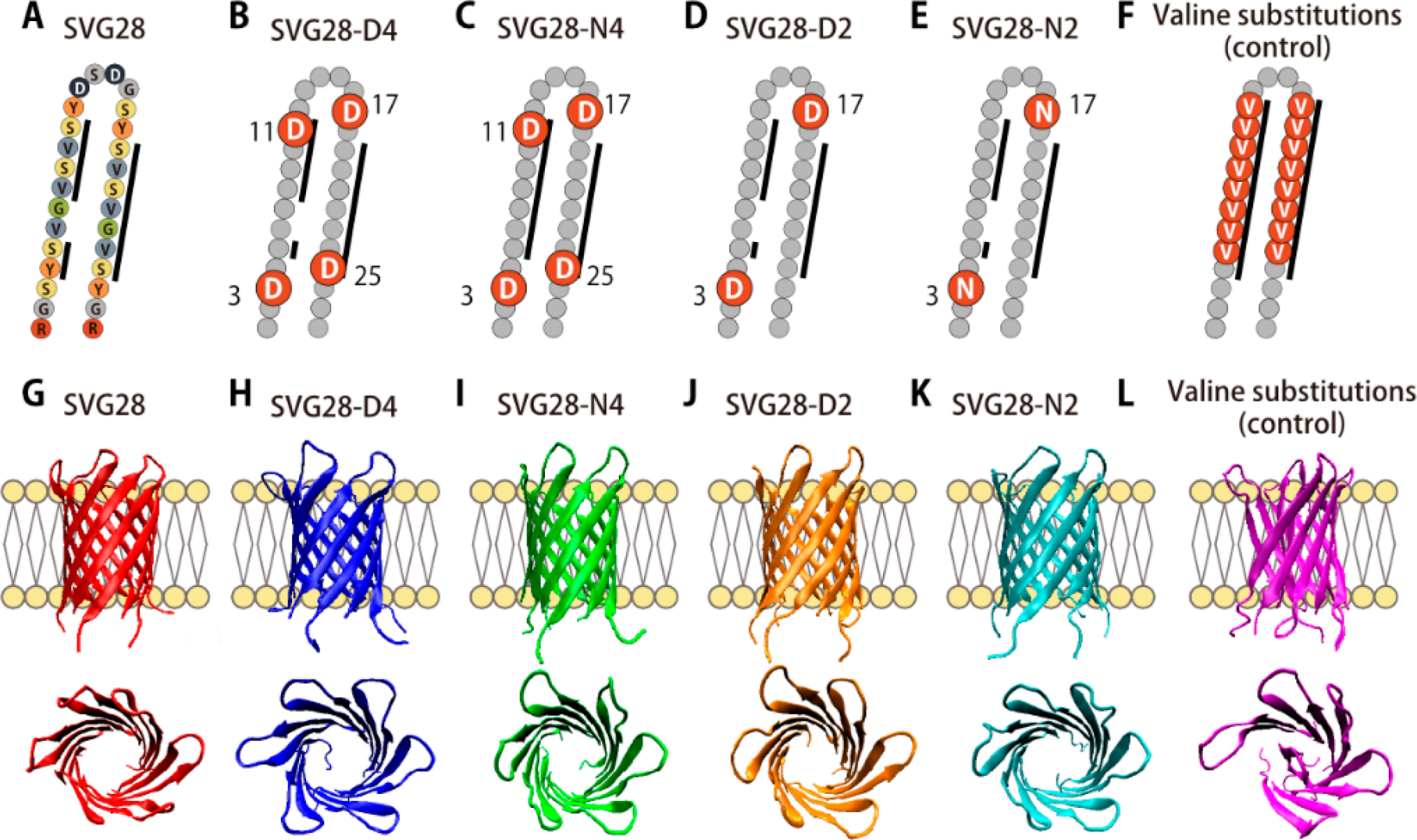
Hydrophilic variants of SVG28. (A–F) Amino acid sequences of SVG28 (A) and the position of hydrophilic mutations in SVG28-D4 (B), SVG28-N4 (C), SVG28-D2 (D), and SVG28-N2 (E) and the control with valine substitutions in the transmembrane region (F). The β-sheet region predicted by MINNOU^43^ is highlighted by black lines. (G–L) Side and top view of final structure of 6-mer SVG28 (G), SVG28-D4 (H), SVG28-N4 (I), SVG28-D2 (J), SVG28-N2 (K), and the control (L) after 200 ns simulation. SVG28-D4, N4, D2, N2 and the control have a starting methionine for cell-free synthesis.

We initially investigated the cell-free synthesis of the most hydrophobic peptide, SVG28 (Figure 1A), as we previously reported.^34^ The synthesis of SVG28 was not confirmed using a MALDI-TOF/MS measurement (Figure S3). The sequence of SVG28 is suggested to be too hydrophobic to obtain enough yield with the cell-free system, or to purify without aggregation. To synthesize peptides using the cell-free system, we redesigned four different types of more hydrophilic variants of SVG28 which were mutated from Ser to Asp or Asn. Asp (D, hydrophobicity: −3.50) and Asn (N, −3.50) are more hydrophilic residues than that of Ser (S, −0.80)^41^ and have relatively compact side chains compared to other hydrophilic amino acids. The four-residue mutated variants named SVG28-D4 (Figure 1B) and SVG28-N4 (Figure 1C) and the two-residue mutated variants named SVG28-D2 (Figure 1D) and SVG28-N2 (Figure 1E) were designed. The hydrophilic residues were introduced at the edge of the β-sheet region to prevent its structure collapse and to avoid the increase of pore noise which would result from adding amino acid residues in the loop and terminal regions.^42^ To design SVG28-D2 and SVG28-N2, two hydrophilic residues at the interior side of the pore were removed from the SVG28-D4 and SVG28-N4 structures, respectively.

We performed all-atom molecular dynamics (MD) simulations to confirm whether the barreled structure is stable in the lipid membrane. The hexametric structure of each peptide was embedded in a system with the same lipid composition and salt concentration as in the experimental study. The pore structure of each variant was retained for 200 ns (Figure 1G–K) compared to the control structure with all-valine substitution in the transmembrane region (Figure 1F,L). The detailed analysis such as the root-mean-square distance (RMSD), root-mean-square fluctuation (RMSF), and other control structures are shown in Figure S4 and S5.

### Cell-Free Synthesis and Confirmation of SVG28-D4, SVG28-N4, SVG28-D2, and SVG28-N2

**Figure 2:**
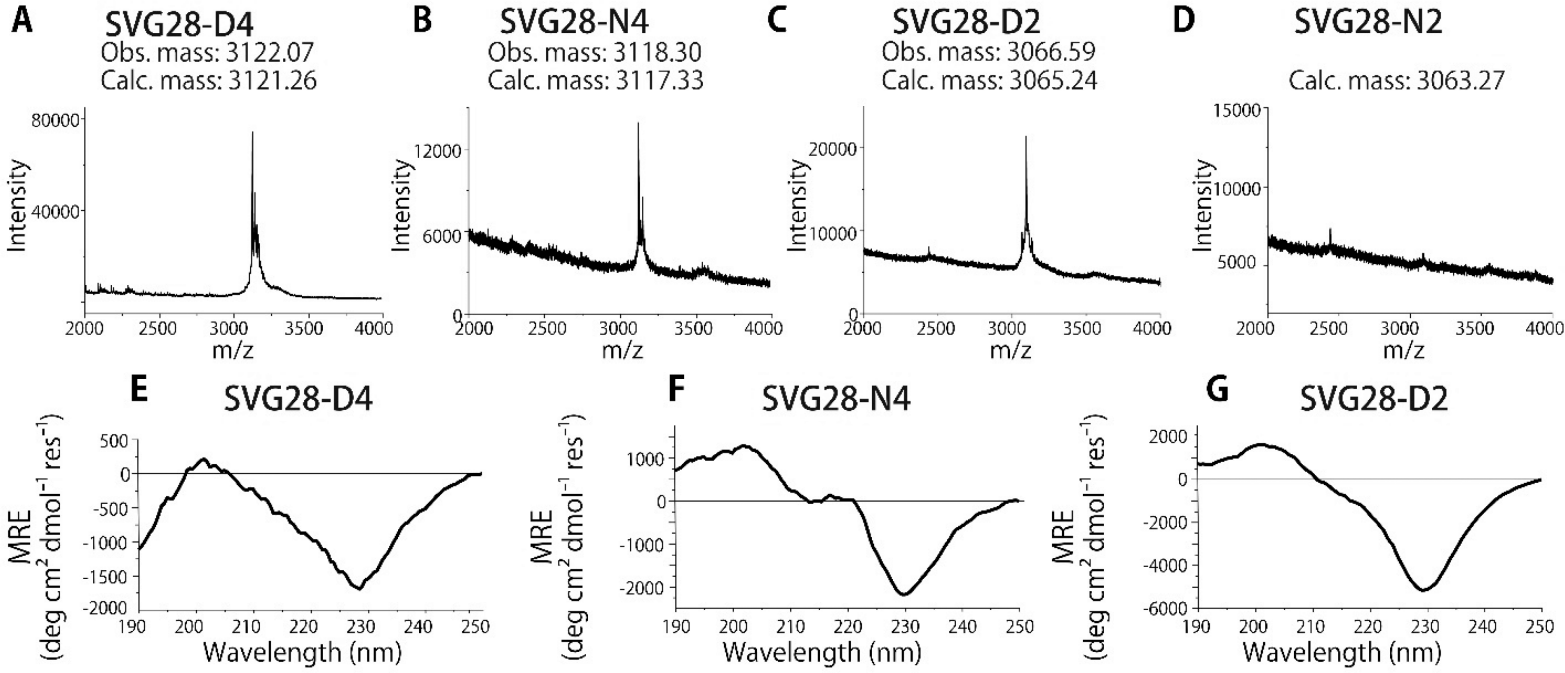
Confirmation of peptide expression. (A–D) MALDI-TOF/MS mass spectra of SVG28-D4 (A), SVG28-N4 (B), SVG28-D2 (C), and SVG28-N2 (D) expressed using cell-free synthesis. Obs. mass and Calc. mass stand for observed and calculated [M + H]^+^ values of the peptides. (E–G) CD spectra of SVG28-D4 (E), SVG28-N4 (F), and SVG28-D2 (G).

A reconstituted cell-free synthesis system (PURE system) was used in this study because it does not contain proteases that degrade the peptide chain.^39,40^ We designed *E. coli* codon-optimized synthetic genes of the mutants (Supporting Information) and tried to express them using the PURE system. MALDI-TOF/MS measurements confirmed the expression of SVG28-D4, N4, and D2 (Figure 2A–C), but not SVG28-N2 (Figure 2D). Although the hydrophobicity index of Kyte and Doolittle,^41^ based on experimental data, indicates the same hydrophobicity for Asp and Asn, only SVG28-D2 of the two-residue mutated variants was successfully synthesized. However, the hydrophobicity index of Engelman predicts that transmembrane α-helical structures have different hydrophobicity for Asn and Asp.^44^ This suggests that there was in fact a hydrophobicity difference between SVG28-D2 and SVG28-N2 in this synthesis.

The secondary structure was confirmed using circular dichroism (CD) spectroscopy. Figure 2E–G suggests formation of β-sheet-like structures in SVG28-D4, N4, and D2 with 0.2 mM *n-*dodecyl-β-d-maltoside (DDM). The expressed amount of each peptide was estimated by UV–Vis spectroscopy (Table S1, Figure S6), and these yields of peptides were sufficient for a channel current measurement using our lipid bilayer system, which only requires a small amount of sample. The amount of expressed peptides was almost equal to that of green fluorescent protein (GFP) and dihydrofolate reductase (DHFR) expressed using the PURE system (Table S2).^45,46^

### Electrophysiological Measurements and Evaluation of the Pore-Formation

**Figure 3:**
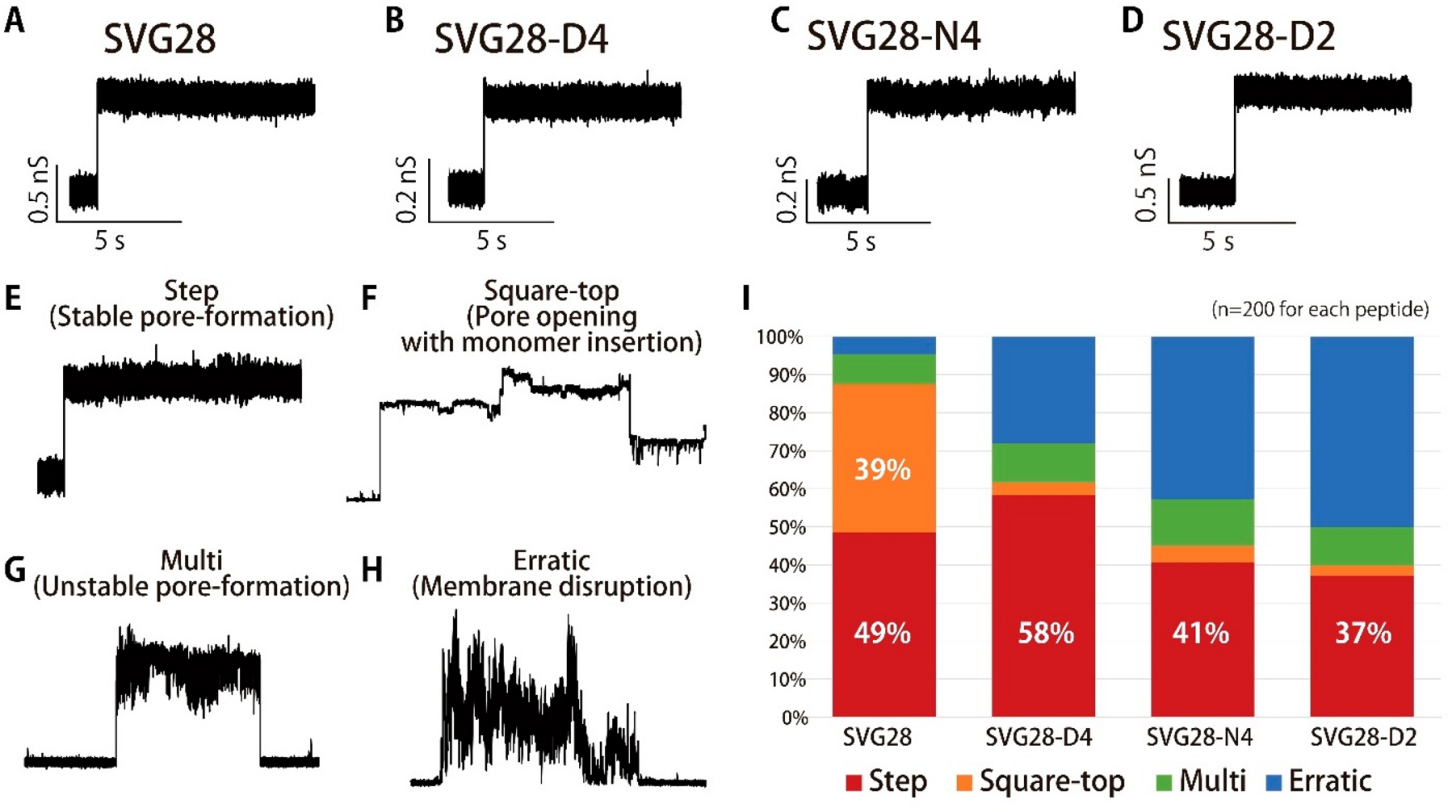
Analysis of signals obtained from SVG28, SVG28-D4, SVG28-N4, and SVG28-D2. (A–D) The typical current–time trace of SVG28 synthesized by solid-phase synthesis (A) and SVG28-D4 (B), SVG28-N4 (C), SVG28-D2 (D) synthesized by cell-free synthesis. (E,F) Step (E) and square-top (F) signals were defined as corresponding to stable pore formation. (G,H) Multi (G) and erratic (H) signals were defined as corresponding to unstable pore formation. (I) The ratio of step, square-top, multi, and erratic signals from the channel current analysis. SVG28 used here was synthesized by solid-phase synthesis.

The pore-formation of SVG28-D4, N4, and D2 were investigated by channel current measurements in the lipid bilayer system. The pore-opening states in which the current momentarily increased were observed for all peptides (Figure 3A–D), indicating that their pore-forming ability was retained despite the introduction of hydrophilic mutations. To determine the probability of opening stable pores, we defined and classified the current signal into four types:^47^ step and square-top signals corresponding to stable pore-formation (Figure 3E,F), and multiple and erratic signals corresponding to unstable pore-formation (Figure 3G,H). The definition of the signal classifications is described in the Supporting Information (Figure S2). The ratio of stable pore-formation for the hydrophilic mutant decreased compared to that of SVG28 synthesized by solid-phase synthesis (Figure 3I and S7). When looking at stable pore-formation, the hydrophilic mutation did not decrease in step signals, associated with single stable pore formation, with 49% of SVG28, 58% of SVG28-D4, 41% of SVG28-N4, and 37% of SVG28-D2 under every voltage application. However, the square-top signals decreased, with 39% of SVG28, 3% of SVG28-D4, 10% of SVG28-N4, and 5% of SVG28-D2 (Figure 3I). The square-top signal would reflect the pore-formation with intermittent monomer insertion and dissociation; therefore, the reduction of the square-top ratio suggests inhibition of the larger pore-formation for these hydrophilic peptides.

**Figure 4:**
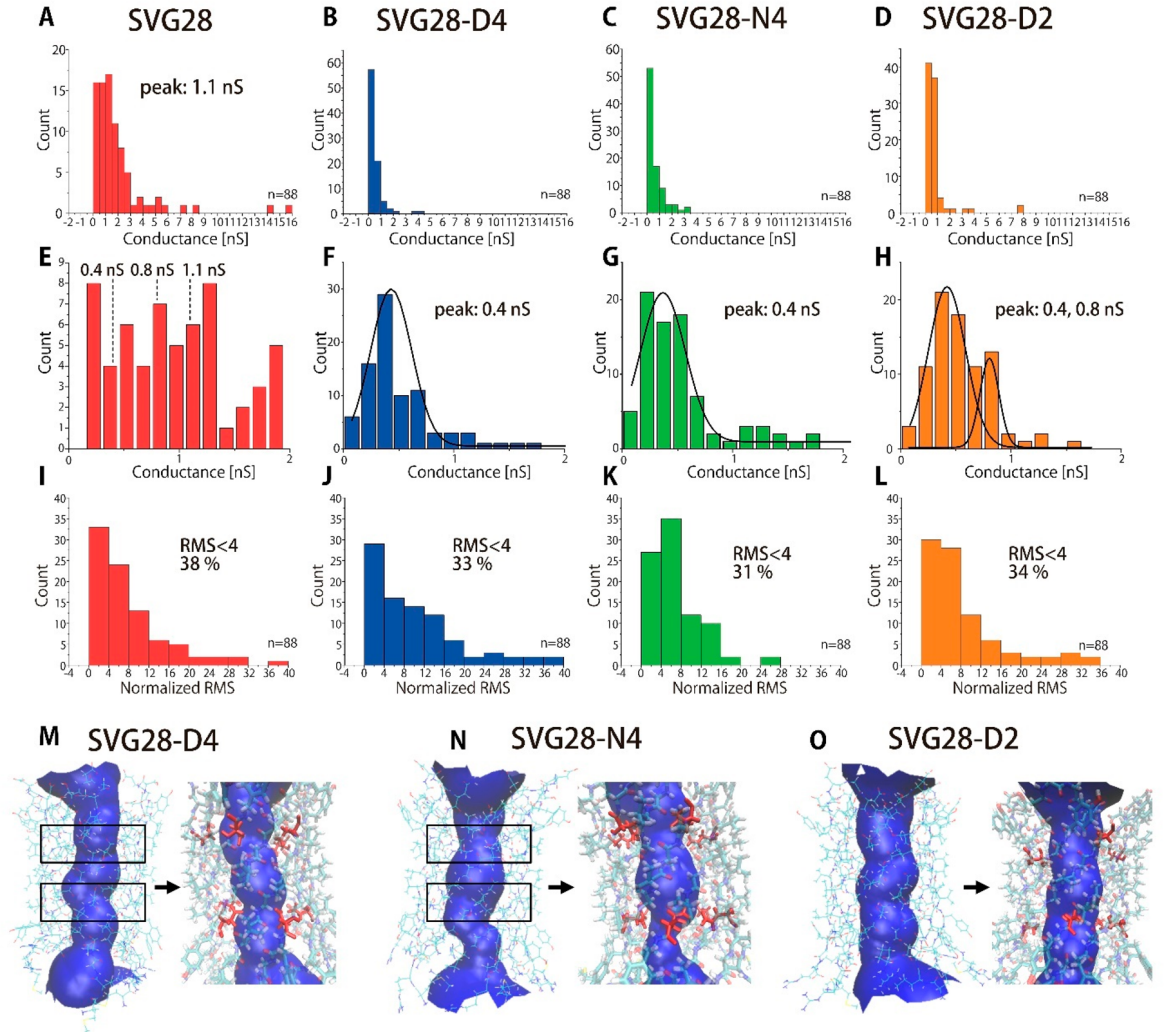
Analysis of pore current of SVG28, SVG28-D4, SVG28-N4, and SVG28-D2. (A–D) Histograms of the current conductance of open levels for SVG28 synthesized by solid-phase synthesis (A), SVG28-D4 (B), SVG28-N4 (C), and SVG28-D2 (D) at +50, 100, 150, and 200 mV. (E–H) The expanded current histogram in the range of 0–2 nS of SVG28 synthesized by solid-phase synthesis (E), SVG28-D4 (F), SVG28-N4 (G), and SVG28-D2 (H). (I–L) The histogram of the normalized RMS noise of SVG28 synthesized by solid-phase synthesis (I), SVG28-D4 (J), SVG28-N4 (K), and SVG28-D2 (L) at all voltages. (M–O) Visualization of the structure of SVG28-D4 (M), SVG28-N4 (N), and SVG28-D2 (O) by HOLE.^49^ The 11th and 17th residues (Asp of SVG28-D4, Asn of SVG28-N4, Ser of SVG28-D2) are visualized in red, showing that the substituted residues generate a constriction in the pentamer pore.

The pore diameter of each nanopore was calculated by the conductance of the initial open-pore current from the baseline level, such as shown in Figure 3A. The histograms of the pore conductance of each peptide are shown in Figure 4A,B and the expanded histograms are shown in Figure 4E–H. The peak conductance of the SVG28-D4 and SVG28-N4 occurred at around 0.4 nS (Figure 4F,G), and that of SVG28-D2 at around 0.4 nS and 0.8 nS (Figure 4H), which were less than that of SVG28 at 1.1 nS^34^ (Figure 4A). We estimated pore size using the relationship between the current conductance and the pore diameter of natural β-barrel proteins as we previously proposed^34^ (Figure S8). The SVG28-D4 and N4 were estimated approximately to have a 5-mer structure with a diameter of ca. 1.1 nm, and the SVG28-D2 was estimated to form 5- and 6-mer structures with a diameter of ca. 1.1 and 1.5 nm, compared to the SVG28 7-mer structure with a diameter of 1.7 nm. The decreased size of the pore and the ratio of square-top signals in the hydrophilic variant suggest that the destabilization of the peptide prevented it from making large and dynamic pore structures, also supporting our proposal of a decrease in the number of monomers.

We next considered the pore noise of the open-pore state. The open state of the step currents seemed to show increased current noise compared to that for the SVG28 pore (Figure S9A). To assess the precise current noise of pores, we attempted to quantify the noise using root-mean-square (RMS) analysis. The inherent RMS noise of a pore current depends on the magnitude of the conductance due to the differences in pore resistance.^29^ In the precise analysis for the different conductance of the signals, we made an approximate curve that was obtained from the plot of RMS noise versus conductance (*n* = 23), and the slope was used for the estimation (Figure S9C). The histogram of the RMS noise is shown in Figure 4I–L, and the law data is presented in Figure S9B for reference. When applying this to the nanopore measurements, we defined the pores with RMS noise of less than 4 to have sufficiently low noise and appropriate for recognition of the translocation of single molecules. The order of the ratio of the RMS with less than 4 was as follows: [SVG28-N4:31%] < [SVG28-D4:33%] < [SVG28-D2:34%] < [SVG28:38%], and overall RMS trends of SVG28-D2 was also improved. This result suggests that the introduction of hydrophilic residues destabilizes the pores in accordance with the number of substitutions. In addition, SVG28-D4 and N4 have the mutation at the inside of the membrane-anchoring tyrosine^48^ (11th and 17th residues), which is positioned at the interface between the hydrophilic lipid head and the hydrophobic lipid tail. The protrusion of bulky hydrophilic residues (Asp and Asn) inside the barrel may have led to increased noise based on the MD simulations (Figure 4M–O).

### Single-Molecule Detection Using the SVG28-D2 Nanopore

**Figure 5:**
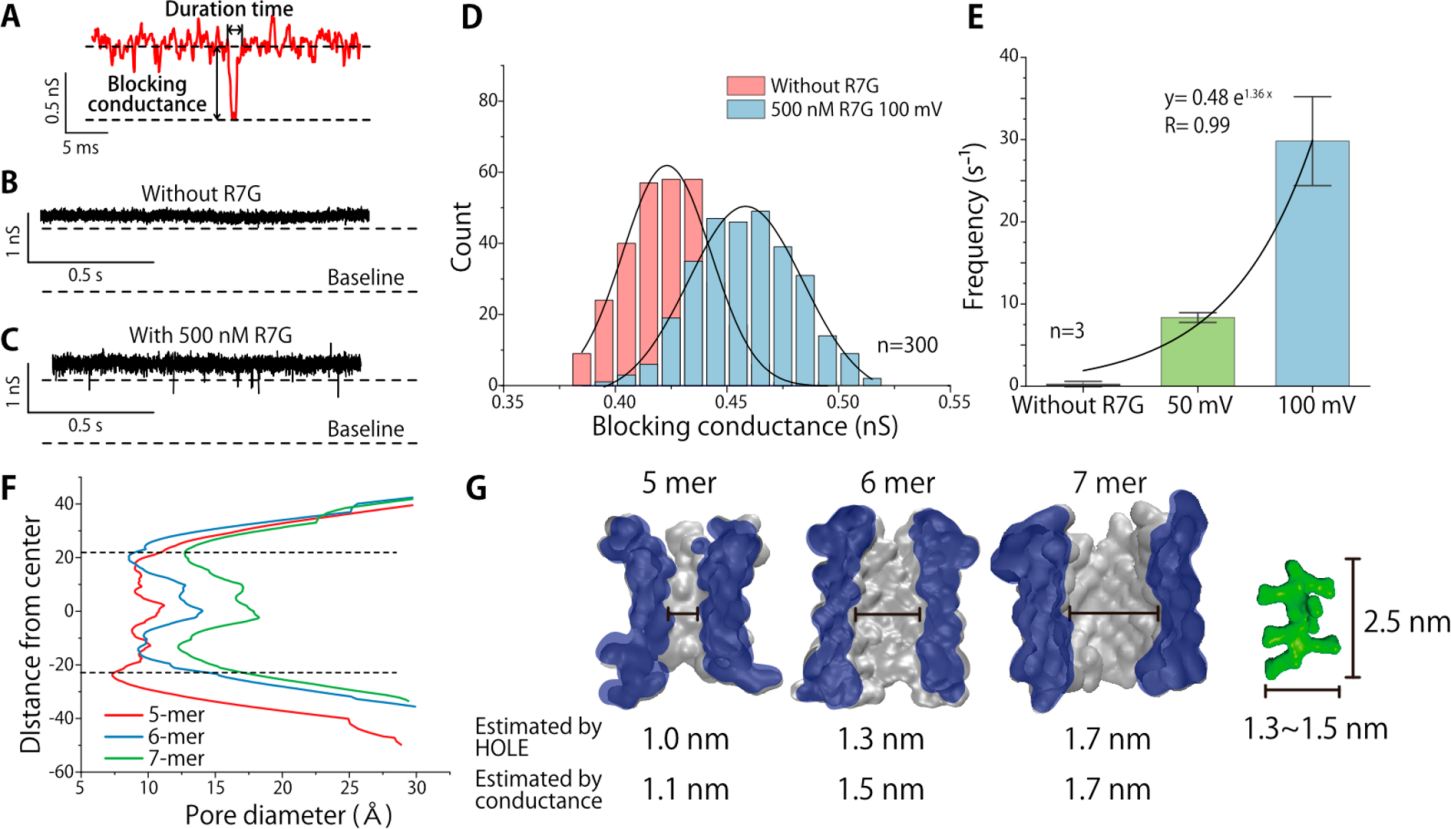
Single-molecule detection. (A) Illustration of a typical blocking current. (B,C) The current and time trace of SVG28-D2 without R7G (B) and with 500 nM R7G (C). The upper dashed lines indicate the threshold for the R7G translocation events and lower lines indicate the baseline (≅0 A). (D) Histogram of the blocking current after bootstrapping of peptide without R7G (control, red, 0.42 nS) and with R7G under 100 mV (green, 0.46 nS). (E) The event frequency of the R7G translocation as a function of the applied voltage. The error bars show the mean ± SE. (F) Pore diameter at each *z*-axis of the 5-, 6-, and 7-mer pore calculated using HOLE. The dashed lines indicate approximate pore areas. (G) Cross section of the pore of 5-, 6-, and 7-mer SVG28. Average pore diameter was calculated using HOLE^49^ and the peak conductance. The R7G structure was predicted by Alphafold2.^50^

The SVG28-D2 formed relatively large pores and had low noise, and thus we attempted use it to detect an oligopeptide. In a previous study, 13 of the 20 natural amino acids positioned at the N-terminus of a heptaarginine (R7-X) were identified using the aerolysin nanopore.^24^ To test the detection capabilities of SVG28-D2, we used RRRRRRRG (R7G) as the target. The SVG28-D2 mainly forms a nanopore with conductance at around 0.8 nS (Figure 4D) which we estimate to have a diameter of ca. 1.5 nm. Although the blocking signal was not observed in the nanopore with this conductance, clear blocking signals (Figure 5A) appeared in the case of much larger nanopores with conductance at around 2 nS (Figure 5B,C). Because SVG28-D2 sometimes shows intrinsic blocking-like noise signals, the precise identification of the signals from the translocation of peptides was a challenge. We compared the blocking amplitudes between the experiments with and without R7G, and investigated the event frequency of the blocking signals against voltage applications. As a result, the histogram of the blocking conductance provides the discrimination of the blocking signal and the pore-derived noise (Figure 5D and S10), and the frequency of blocking events increased with an increase in applied voltage, suggesting that the blocking signals reflect the translocation of R7G (Figure 5E). Although the large distribution in signal duration makes it difficult to strictly discuss, the duration time of translocation signal under 100 mV was slightly longer than under 50 mV. See Figure S10 for details.

In order to detect R7G, a nanopore with diameter much larger than ca. 1.3 nm is required, based on the HOLE analysis of MD simulations in this experiment (Figure 5F,G). The results of the blocking signal observed only at higher conductance also indicates that the decreased conductance of hydrophilic variants was due to a decreasing number of monomers. The aerolysin nanopore previously used for the detection of the same oligopeptide has at its narrowest a 1.0 nm constriction calculated by HOLE analysis, suggesting that a larger vestibule region for capturing a target molecule is needed for frequent nanopore-sensing with narrow constrictions. Moreover, the upper three negative charges per monomer in SVG28-D2 have been suggested to prevent the insertion of the cationic peptide into the pore, compared to the detection using SVG28 (data not shown), and the reduction of one positive charge at the pore exit have also contributed to the faster translocation of the oligopeptide.

**Figure 6:**
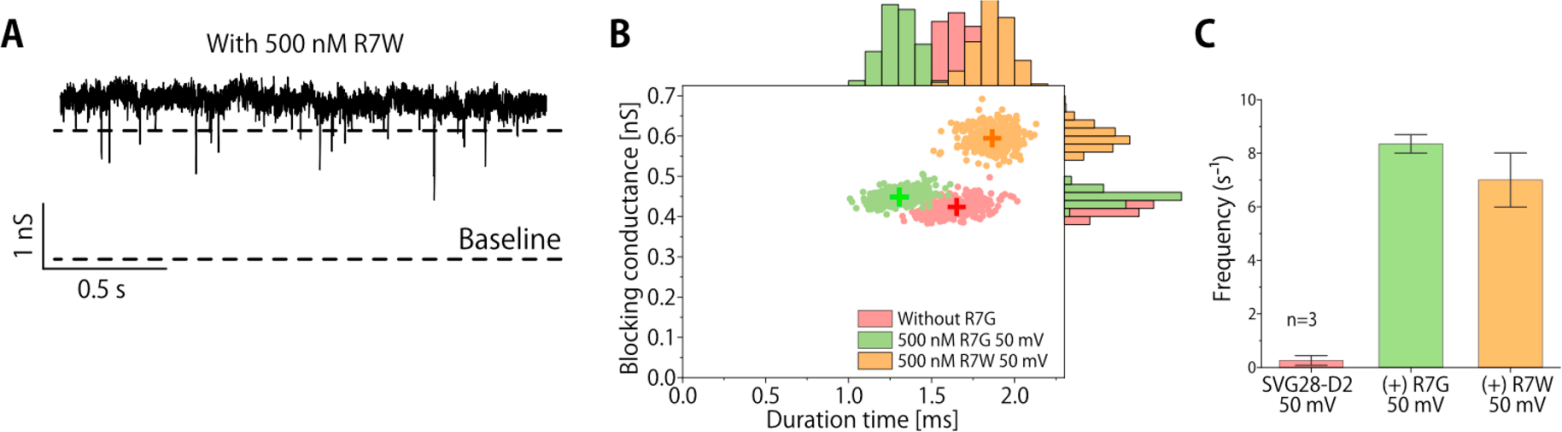
Single-molecule detection of another peptide, R7W. (A) The current and time trace of SVG28-D2 with 500 nM R7W. The upper dashed lines indicate the threshold for the R7W translocation events and lower lines indicate the baseline (≅0 A). (B) Scatter plots of blocking current and duration time after bootstrapping of SVG28-D2 without R7G (control, red, 1.65 ms, 0.42 nS), with R7G under 50 mV (green, 1.30 ms, 0.45 nS), and R7W under 50 mV (orange, 1.86 ms, 0.59 nS) The center of the plot is represented by crosses. (C) The event frequency of the noise of SVG28-D2 and the R7G and R7W translocation as a function of the applied voltage. The error bars show the mean ± SE.

As in the previous study of aerolysin,^24^ the change in the blocking amplitude depending on the side chain volume was examined using RRRRRRRW(R7W), which has a larger side chain than R7G. Clear blocking signals were observed only with ca. 2 nS pore, similar to the single-molecule measurement of R7G (Figure 6A). The blocking conductance of R7W was clearly discriminated from pore-derived noise (Figure 6B), and the frequency of blocking events increased compared to pore noise (Figure 6C), suggesting the blocking signals reflect the translocation of R7W. The blocking amplitude was increased with the increase of the volume of the side chain (Figure 6B), indicating that the SVG28-D2 nanopore has the potential for peptide discrimination at one amino acid resolution, although this is not a measurement of its main conductance.

## Conclusion

We used a cell-free system to express hydrophilic mutants of the SVG28 peptide and establish a method for exploring the systematic sequence design of peptide nanopores. The hydrophilic variants were designed with two or four substitutions from Ser to Asp or Asn. A surprising result was that the expressed variants retained the pore-forming ability despite the substitution of four (about 14%) and two (about 7%) of the total of 28 residues with more hydrophilic amino acids. The pore conductance of the variants converged less than that of SVG28, and the amount of pore noise increased according to the number of hydrophilic mutations. The changes in conductance and pore noise due to the introduction of the mutations provides insight into the assembly state of β-barrels formed by the association of β-hairpins and provides knowledge that can be applied in nanopore design. Single-molecule detection of oligopeptides and discrimination of them at one amino acid resolution were achieved with a ca. 2 nS nanopore, but not with the main conductance of SVG28-D2. However, through this sequencing experiment we confirmed that our intended outcomes were successful in the design and fabrication of cell-free synthesizable nanopores. Although previous synthesis of nanopore proteins using a cell-free system and with the addition of liposomes has been reported,^51,52^ our cell-free synthesizable peptide nanopores do not require lipid components and the pore properties can be easily modulated through sequence modification, thus enabling the easy custom design of nanopore sequences and comprehensive sequence screening of *de novo* nanopore-forming β-hairpin peptides.

## Methods

### Reagents and Chemicals

The following reagents were used: Purefrex2.0 (GeneFrontier, Japan); GL-Tip SDB (GL Sciences, Japan); Trifluoroacetic acid (TFA, FUJIFILM Wako Pure Chemical Industries, Ltd. (Wako), Japan); acetonitrile (ACN, FUJIFILM Wako Pure Chemical Industries, Ltd. (Wako), Japan), α-cyano-4-hydroxycinnamic acid (CHCA, Tokyo Chemical Industry, Japan); polyethylene Glycol 4,000 (PEG4000, Tokyo Chemical Industry, Japan); *n*-dodecyl-β-d-maltoside (DDM, FUJIFILM Wako Pure Chemical Industries, Ltd. (Wako), Japan); 1,2-dioleoyl-*sn*-glycero-3-phosphocholine (DOPC, Avanti Polar Lipids, USA); cholesterol (Sigma-Aldrich, USA); *n*-decane (FUJIFILM Wako Pure Chemical Industries, Ltd. (Wako), Japan); 3-morpholinopropane-1-sulfonic acid (MOPS, Nacalai Tesque, Japan); potassium chloride (KCl, Nacalai Tesque, Japan); potassium hydroxide (KOH, FUJIFILM Wako Pure Chemical Industries, Ltd. (Wako), Japan); dimethyl sulfoxide (DMSO, FUJIFILM Wako Pure Chemical Industries, Ltd. (Wako), Japan). SVG28 was synthesized and purified by Fmoc synthesis. DNA templates for cell-free synthesis were synthetic genes provided by Integrated DNA Technologies, USA. DOPC was diluted to 10 mg/mL in *n*-decane. Buffered electrolyte solutions were prepared from ultrapure water, which was obtained from a Milli-Q system (Millipore, Billerica, USA). SVG28 was dissolved at a concentration of 100 μM in ultrapure water:DMSO = 1:1 (v/v) and stored at −30 °C. The solid-phase synthesis of SVG28 and confirmation of synthesis is described in our previous paper.^34^ Products of cell-free synthesis were dissolved at a concentration of 10 μM in ultrapure water and stored at −30 °C.

### Molecular Dynamics Simulations

All-atom molecular dynamics (MD) simulations of the β-barrel structure of the SVG28, SVG28-D4, SVG28-N4, SVG28-D2, and SVG28-N2 were performed to confirm the pore-forming ability of each peptide in the bilayer lipid membrane. The 3D initial structures of each peptide were modeled via a homology modeling technique using SWISS-MODEL.^53^ HASR protein (PDB ID: 3CSL; chain A; 484–511) was selected as a template structure for the peptides, in keeping with our previous study. The obtained monomeric structure was carefully aligned into 4-, 5-, 6-, and 7-mer structures, which were used as the master structure. Alignment of each monomer to the master structure was performed by USCF Chimera.^54^ Each polymeric structure was used as the starting structure and the remaining components were prepared by the CHARMM-GUI membrane builder.^55^ Each polymeric structure was embedded in DOPC:Cholesterol = 2:1 (99 lipids per leaflet) with 1 M KCl at 298 K. Systems were initially equilibrated using the protocol provided by CHARMM-GUI followed by an additional 500 ps of equilibration to allow for the system to fully relax, with a total simulation time of 200 ns. All simulations were executed with GROMACS-2021.2^56^ using the CHARMM36m force field^57^ and run on a computer with Ryzen7 CPU and RTX3070 GPU. The trajectory file was analyzed using the standard analysis tools installed in GROMACS. HOLE software^49^ was used to evaluate the pore diameter in the trajectory.

### Cell-Free Synthesis of β-Hairpin Peptides

Purefrex2.0 (GeneFrontier, Japan), a reconstituted cell-free synthesis system,^40^ was used for cell-free synthesis. Template DNAs for the translation were synthesized to contain the T7 promoter sequence, ribosome binding site (SD sequence), target protein sequence and terminator (Supporting Information). The template DNAs were dissolved at a concentration of 10 ng/μL in DNase-free water (Millipore, USA) and stored at 4 °C. Expression was performed according to the protocol provided at 37 °C for 6 h on a heat block, and 1 μL of DNA template diluted to 0.3 ng/μL was added to the reaction mixture. After the translation, the peptides were desalted with GL-tip SDB (GL Sciences, Japan). The washing solution was 0.1% (v/v) TFA and the eluting solution was 80% (v/v) ACN, 20% (v/v) 0.1% TFA. At first, the tip column was conditioned with 20 μL of eluting solution (3000*g*, 2 min) and then equilibrated with 20 μL of washing solution (3000*g*, 2 min). After that, the reaction mixture was added to the column and centrifuged (3000*g*, 5 min) and the column was washed with 20 μL of washing solution (3000*g*, 2 min). Finally, 100 μL of eluting solution was added to the column, centrifuged (3000*g*, 3 min), and collected in a collection tube. Then the acetonitrile in the desalted solution was removed under a vacuum, and the remaining sample was lyophilized using a Freezone 6-PLUS (Asahi Life Science Corporation, Japan).

Matrix-assisted laser desorption or ionization with time-of-flight (MALDI-TOF) mass spectroscopy analyses were carried out using the MALDI-TOF autoflex speed (Bruker, USA) in linear/positive mode. PEG4000 was used for external mass calibration. The lyophilized peptides for 20 μL of the reaction mixture were dissolved in 3 μL of TA30 (ACN:0.1% TFA = 3:7, v/v). 100 μg α-cyano-4-hydroxycinnamic acid (CHCA) dissolved in 100 μL TA30 and the peptide sample were mixed in a sample–matrix ratio of 1:2 (v/v), and 1 μL of the solution was dropped on the plate. To confirm the β-hairpin structure of the expressed peptide, CD (circular dichroism) measurements were performed using a J-1100 CD Spectrometer (JASCO, Japan). The lyophilized peptides for 20 μL of the reaction mixture were dissolved in 2 μL of 0.2 mM DDM and 1 mM MOPS. A quartz microsampling disc with a 0.2 mm path length was used for the measurements. The spectra were acquired every 0.5 nm from 190 to 300 nm with 20 mdeg/0.05 dOD of CD scale, 1 nm of bandwidth, and four times integration. Molar residue ellipticity (MRE [deg cm^2^ dmol^–1^ res^–1^]) was calculated from the observed ellipticity (mdeg) by the following equation:
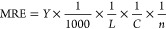
MRE: Molar Residue Ellipticity [deg cm^2^ dmol^–1^ res^–1^], *Y*: observed ellipticity [mdeg], *L*: path length [cm] *C*: peptide concentration [dmol cm^–3^] *n*: residue length of peptide. For each data set, baselines from the same buffer and sampling disc were subtracted, and the observed ellipticity of the purified and lyophilized cell-free solution (by replacing the DNA solution with purified water) was also subtracted. The signal was smoothed by the Savitzky–Golay method with a convolution width of 15.

To confirm the amount of expressed peptide, absorbance measurements were carried out using a Nanodrop 2000c (Thermo Scientific, USA) in UV–Vis mode. The absorption at 205 nm derived from the peptide bond is used for the estimation of peptide concentration. The absorption coefficient of each peptide was calculated from the amino acid sequence of the peptide.^58^ Each peptide solution was aliquoted to 10 μM in 20 μL and stored at −30 °C.

### Preparation of Microdevice for BLM Formation

The microdevice was fabricated by machining a 6.0 nm thick, 10 × 10 mm poly(methyl methacrylate) (PMMA) plate (Mitsubisi Rayon, Japan) using a computer-aided design and computer-aided manufacturing three-dimensional modeling machine (MM-100, Modia Systems, Japan) as shown in Figure S1A. Two wells (2.0 nm diameter and 4.5 nm depth) and a chase between the wells were manufactured on the PMMA plate (Figure S1B). Each well had a through-hole in the bottom and Ag/AgCl electrodes set into these holes (Figure S1A). A polymeric film made of parylene C (polychloro-*p*-xylylene, Parylene Japan, Japan) with a thickness of 5 μm was patterned with single pore (100 μm diameter) using the conventional photolithography method, and then put between PMMA sheets (0.2 mm thick) (Figure S1C). The sheets enclosing the parylene C film were inserted into the chase to separate the two chambers.

### Bilayer Lipid Membrane Preparation Using the Microfabricated Device

Bilayer lipid membranes (BLMs) were prepared using the drop contact method in the microdevice. In the drop contact method as we have been developing,^59−65^ the two lipid monolayers containing the aqueous droplets come into contact in a small hole punched in the parylene film to form lipid bilayers (Figure S1D). Lipid bilayers were formed as follows: first, 0.6 μL of DOPC:Cholesterol = 4:1 (w/w) dissolved in *n*-decane was added into both chambers. Then 4.7 μL of 1 M KCl and 10 mM MOPS was added to a chamber on the voltage applied side and 4.7 μL of 1 M KCl, 10 mM MOPS, and 1 μM SVG or 5 μM SVG28-D4, SVG28-N4, or SVG28-D2 was added to a chamber on the ground side. When the BLM was broken, a thin hydrophobic stick was used to trace back the lipid and reconstitute the BLM.

### Channel Current Measurements

The channel current was monitored using a JET patch clamp amplifier (Tecella, USA). Signals were detected using a 4 kHz lowpass filter at sampling frequency of 20 kHz. A constant voltage of +50, +100, +150, and +200 mV was applied to the recording chamber, and the chamber on the ground side was grounded. Data were analyzed using Clampfit 11.1 (Molecular Devices, USA), Excel (Microsoft, USA), OriginPro 2022 (Light Store, Japan), and Python 3.9 (Python Software Foundation, USA). The channel current measurement was performed at 22 ± 2 °C.

### Current Signal Analysis of the Peptide Nanopores

To evaluate the pore-forming ability of the peptides, current signals were classified into stable pore-formation signals and unstable pore-formation signals, using the signal classification method previously proposed in our laboratory^47^ and used for SVG28.^34^ The current signals were classified into four types: step, square-top, multi, and erratic. The step signal, representing stable single pore formation, shows a sharp signal rise in less than 10 ms, and the 95% confidence interval of the pore current value less than 10 times that of the baseline. A signal lasting longer than 1 s was classified as a step signal (Figure S2A). The square-top signal, representing stable pore formation involving insertion and dissociation of monomers, shows a sharp signal rise in less than 10 ms and shoes a staircase current, with the 95% confidence interval of the pore current value less than 10 times that of the baseline for each step (Figure S2B). The multi signal, representing unstable pore formation, shows a sharp signal rise in less than 10 ms, and the 95% confidence interval of the pore current value is more than 10 times that of the baseline (Figure S2C). The erratic signal, representing membrane disruption, shows a blunt signal rise in more than 10 ms (Figure S2D). In addition, signals longer than 1 s were included in the analysis because the hydrophilic variants showed many short duration signals of less than 1 s. The signal distribution was calculated by taking *n* = 20 data of the obtained signal from each measurement, in the order of the longest signal duration, and *n* = 50 data of the sum of them, in the order of the longest duration. The pore conductance was calculated by dividing the current value of the step signal by the applied voltage. The current value is the average of the current values for 1 s after pore opening. To estimate the pore diameter and the number of monomers, the relationship between the conductance of the natural β-barrel proteins CymA, OmpA, OmpF, OmpG, FhuA, and Vdac and the reported pore diameters^34^ was used for the calculation (Figure S8), instead of the commonly used Hille’s equation.^66^ To quantify the noise in the step signal in detail, the RMS noise was calculated by subtracting the unbiased variance of the baseline for approximately 1 s before the pore opening, from the unbiased variance of the current value for approximately 1 s after the pore opening, and taking the square root of the result. Since this value tends to increase in response to pore conductance, an approximate straight line was drawn using all conductance and RMS data (*n* = 352) and normalized to the RMS when the pore was at 1 nS.

### Solid-Phase Synthesis of R7X Peptide

R7G and R7W peptides were synthesized by standard 9-fluorenylmethoxycarbonyl (Fmoc)-based solid phase peptide method using *N*-α-(9-fluorenylmethoxycarbonyl)-glycine and tryptophan *p*-methoxybenzyl alcohol resins (Fmoc-Gly-alko and Fmoc-Trp-alko resins) in the following procedure. To a swollen Fmoc-X-alko resin (0.25 mmol/g) in NMP using 10 mL PTFE reactor vial were added *N*-α-(9-fluorenylmethoxycarbonyl)-*N*-ω-(2,2,4,6,7-pentamethyldihydrobenzofuran-5-sulfonyl)-l-Arginine (Fmoc-l-Arg(Pbf)-OH, 4 equiv), 1,3-diisopropylcarbodiimide (DIC, 4 equiv), and 1-hydroxybenzotriazole monohydrate (HOBt, 0.5 M, 4 equiv) and the reaction mixtures were stirred for 5 min at 75 °C using microwave-assisted peptide synthesizer (Biotage Initiator+ Alstra). Fmoc groups were removed by 20% piperidine in NMP. The resins were filtered, washed 3 times using dichloromethane (DCM), and dried under a high vacuum. The cleavage from resin and deprotection were performed by stirring with TFA, H_2_O, thioanisole, 1,2-ethanedithiol (35/2/2/1, v/v/v/v) for 120 min. The collected filtrate was evaporated and precipitated with cold ether. Finally, crude peptide was extracted using acetonitrile/pure water (v/v = 7:3) from the filtrate. R7X peptide was purified by reverse phase HPLC H-Class (Waters) with WATERS ACQUITY HSS T3 (C18) 1.8 μm (2.1 × 150); detection: 220 nm, column temperature: 298 K, elution: 0.1% TFA in water and 0.1% TFA in an acetonitrile gradient system. The peaks of R7G and R7W were obtained at retention times of 7.267 and 6.186 min (Figure S11A,B). The purified peptides were obtained after lyophilization with 99.3% (R7G) and 98.5% (R7W) HPLC purity. The product *m*/*z* values were confirmed as 1168.65 [M + H]^+^ and 1297.98 [M + H]^+^ by LC/MS with electrospray ionization mode (Waters H-Class QDa) as a reference of ideal *m*/*z* = 1168.75 [M + H]^+^ and 1297.81 [M + H]^+^ (Figure S11C,D).

### Single-Molecule Detection of the Peptide Nanopores

4.7 μL of 1 M KCl, 10 mM MOPS and 500 nM NH_2_-RRRRRRRG-COOH (R7G) or NH_2_-RRRRRRRW-COOH (R7W) was added to the chamber on the voltage-applied-side and 4.7 μL of 1 M KCl, 10 mM MOPS and 5 μM SVG28-D2 was added to the ground side. A constant voltage of +50 or +100 mV was applied to the recording chamber, and the chamber on the ground side was grounded. Because the SVG28-D2 nanopore with 2 nS almost shows noise-like signal longer than 3 nS (78%), the data were filtered to its duration <3 ms to extract the translocation signal. Since the number of available noise signals of SVG28-D2 with 2 nS is too small for valid comparison, signals with 1 nS pore were used as control. The duration time of the translocation signal is the length of time of the current deflected from the mean open pore current (Figure 5A). To avoid including pore noise, the signal is thresholded to take a current value that has decreased beyond a given value. The bootstrap method is a method based on the resampling of the original random sample, drawn from a population with an unknown distribution. We used the exact bootstrap method, which availed of the entire space of resamples. In the exact bootstrap method, accuracy verification is possible when the sample number is greater than 30.^67^ In this study, our bootstrap procedure randomly took 30 samples from the primary common translocation data with 65,536 replacements and calculated the mean for these samples. Three hundred samples were randomly extracted from the bootstrapped data to produce the scatter plot of duration time versus current blocking conductance (Figure S10A).

## Supporting Information Available

DNA sequences of peptides in cell-free expression, detailed information on signal classification, pore analysis and RMS normalization, additional results of MALDI-TOF/MS, MD simulation, UV spectrometry and signal classification and single-molecule detection, yield of each expressed peptide, examples of signals at each RMS value, and confirmation of solid-phase synthesis of R7X peptide (PDF)
