# Supplementary material for: Cell-Free Expression of De Novo Designed Peptides That Form β-Barrel Nanopores
Source: ACS Nano. 2023 Feb 2;17(4):3358–67. doi: 10.1021/acsnano.2c07970 (PMC9979648; doi:10.1021/acsnano.2c07970)
Supplement: Supplementary file 1 — nn2c07970_si_001.pdf [file nn2c07970_si_001.pdf]

Supplementary information

Cell-free expression of *de novo* designed peptides that form β-barrel nanopores

Shoko Fujita[1], Izuru Kawamura[2], and Ryuji Kawano[1]

1. Department of Biotechnology and Life Science, Tokyo University of Agriculture and Technology, Tokyo, 184-8588, Japan

2. Graduate School of Engineering Science, Yokohama National University, Yokohama, 240-8501, Japan.

E-mail (corresponding author): rjkawano@cc.tuat.ac.jp

Supplementary text 1 | Nucleotide sequence for cell-free synthesis of each peptide

SVG28

TAATACGACTCACTATAGGGAGACCACAACGGTTTCCCTCTAGAAATAATTTTGTTTAACTTTAAGA
AGGAGATATCATATGAGAGGTTCATATTCAGTGGGCGTAAGCGTTAGCTACGACTCGGATGGTTCTT
ATAGTGTGAGCGTTGGCGTCTCATATGGGCGCTAATAATGAATAACTAATCC

SVG28-D4

TAATACGACTCACTATAGGGAGACCACAACGGTTTCCCTCTAGAAATAATTTTGTTTAACTTTAAGA
AGGAGATATCATATGAGAGGAGATTATTCAGTGGGCGTTAGTGTAGATTATGATTCCGATGGTGATT
ACTCTGTCTCGGTGGGCGTTGACTACGGTCGCTAATAATGAATAACTAATCC

SVG28-N4

TAATACGACTCACTATAGGGAGACCACAACGGTTTCCCTCTAGAAATAATTTTGTTTAACTTTAAGA
AGGAGATATCATATGAGAGGTAATTATTCAGTGGGCGTAAGCGTGAATTACGATTCGGACGGTAACT
ATAGTGTTTCTGTTGGCGTGAACTACGGCCGTTAATAATGAATAACTAATCC

SVG28-D2

TAATACGACTCACTATAGGGAGACCACAACGGTTTCCCTCTAGAAATAATTTTGTTTAACTTTAAGA
AGGAGATATCATATGAGAGGAGATTATTCAGTGGGGGTAAGCGTTTCGTACGATAGCGATGGTGACT
ATTCAGTCAGTGTGGGCGTTTCCTATGGCCGTTAATAATGAATAACTAATCC

SVG28-N2

TAATACGACTCACTATAGGGAGACCACAACGGTTTCCCTCTAGAAATAATTTTGTTTAACTTTAAGA
AGGAGATATCATATGAGAGGTAATTATTCAGTCGGTGTAAGCGTGTCTTACGATTCGGACGGCAATT
ACTCCGTTAGTGTTGGCGTGTCATATGGGCGCTAATAATGAATAACTAATCC

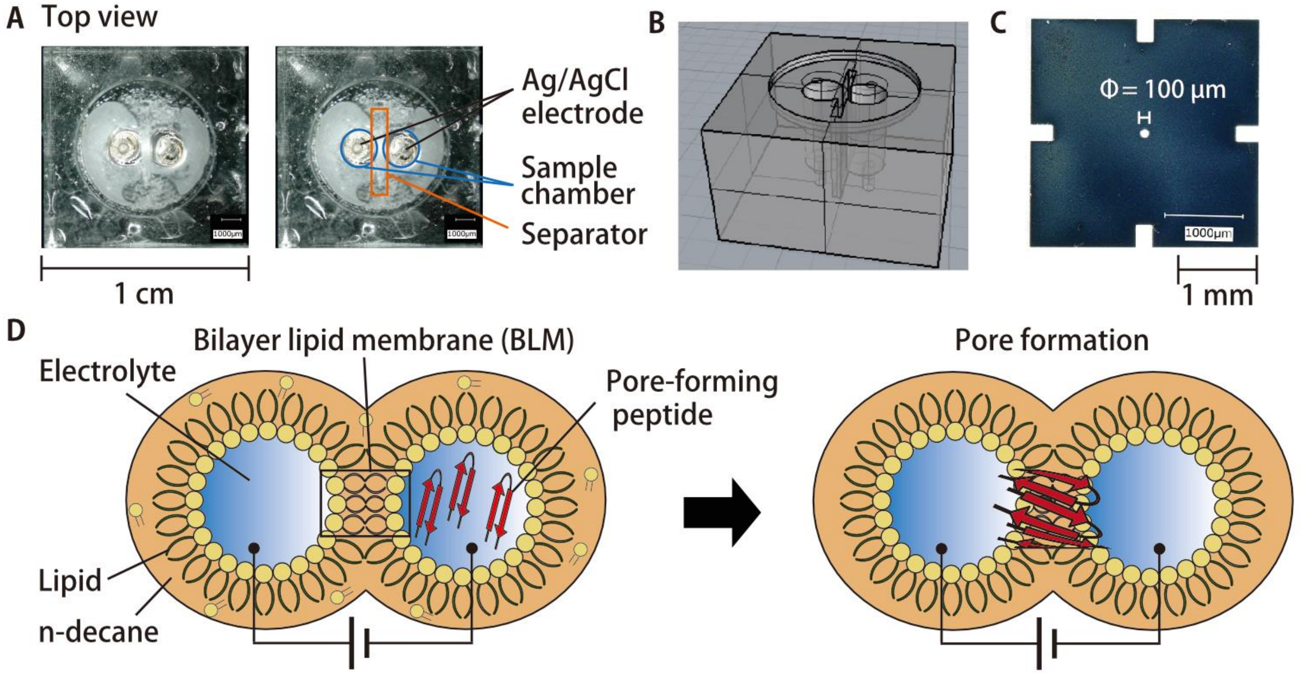

Fig. S1. Configuration of microdevice for channel current measurement. A. A microdevice has two sample chambers with a separator region dividing the chambers and Ag/AgCl electrodes embedded in the bottom of the sample chamber. **B**. 3D image of the structure inside the chamber. Two separators with parylene film sandwiched between them are embedded in the square grooves. **C**. A bilayer lipid membrane is formed in the hole located in the center of parylene film. **D**. Two lipid monolayers containing aqueous droplets come into contact to form the lipid bilayer.

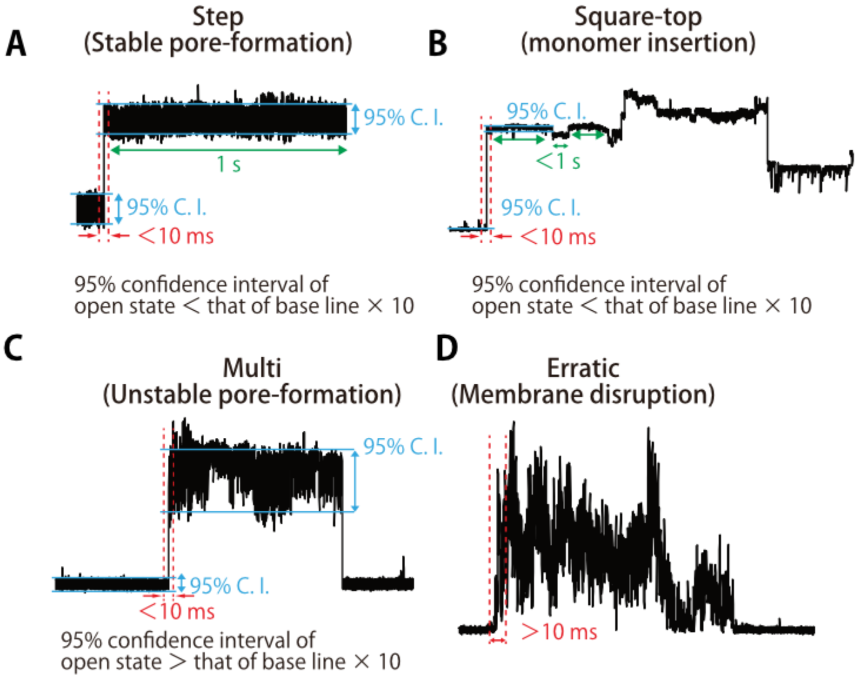

Fig. S2. Detailed criteria for signal classification. A,B. Step (**A**) and Square-top (**B**) signals were defined as stable pore formation. **C,D**. Multi (**C**) and erratic (**D**) signals were defined as unstable pore formation.

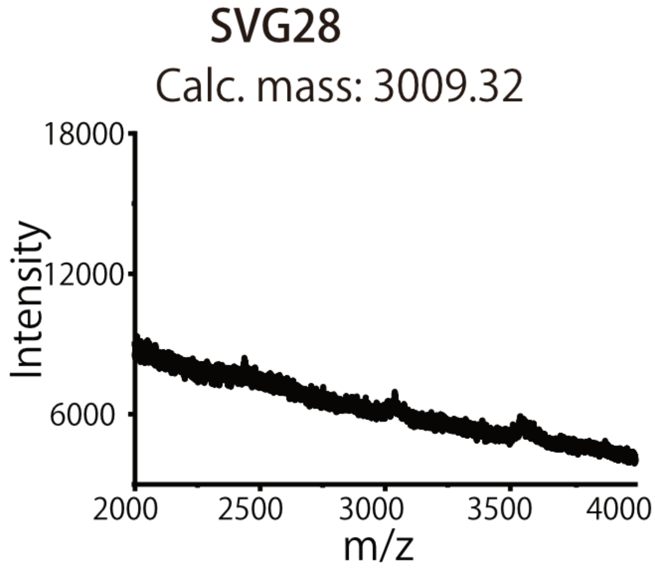

Fig. S3 MALDI-TOF/MS mass spectrum of SVG28 expressed using cell-free synthesis. Peaks corresponding to molecular weights were not observed**.**

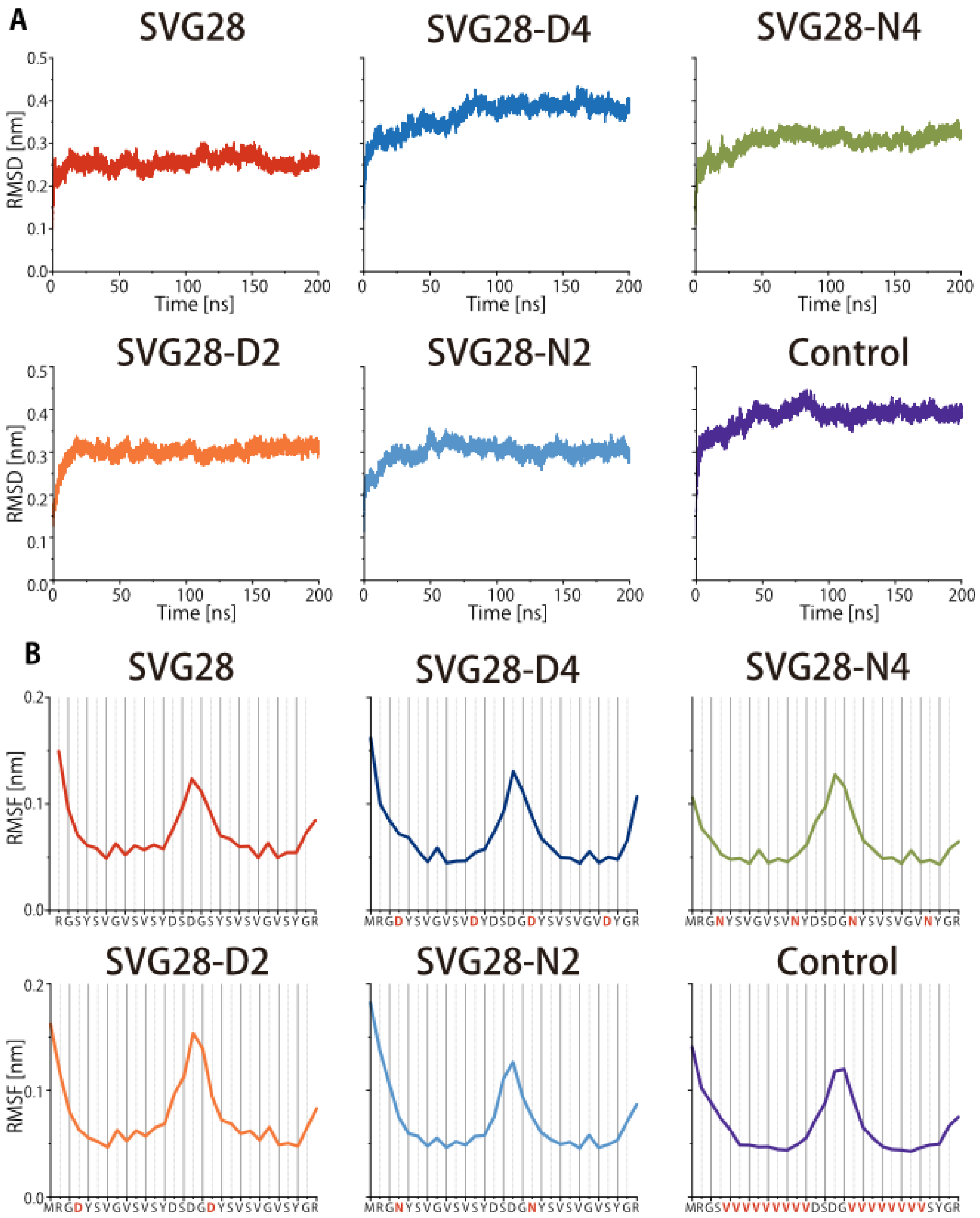

Fig. S4. MD simulation of each peptide. A. Root-mean square distance (RMSD) of each peptide for the entire 200 ns calculation. The RMSD was calculated for the Cα peptide carbonyl. **B**. Root mean square fluctuation of each peptide for the last 20 ns of the 200 ns simulation. RMSF was also calculated for the Cα carbonyl for each peptide residue.

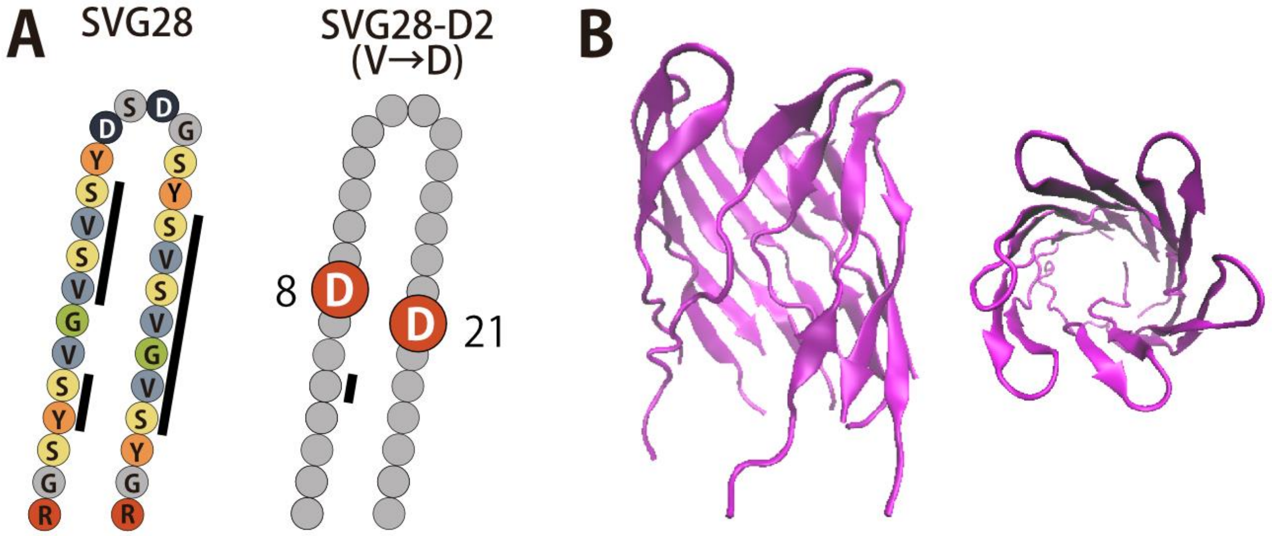

Fig. S5. Another control structure. A. Amino acid sequences of SVG28 and SVG28-D2 with a hydrophilic mutation from Val to Asp. The β-sheet region predicted by MINNOU[52] is highlighted with the black line. **B**. Final structure of 6-mer SVG28-D2 (from Val to Asp) after 200 ns simulation. There is a starting methionine for cell-free synthesis. For sequences predicted to have low β-sheet propensity, their secondary structure was broken in the simulation during 200 ns.

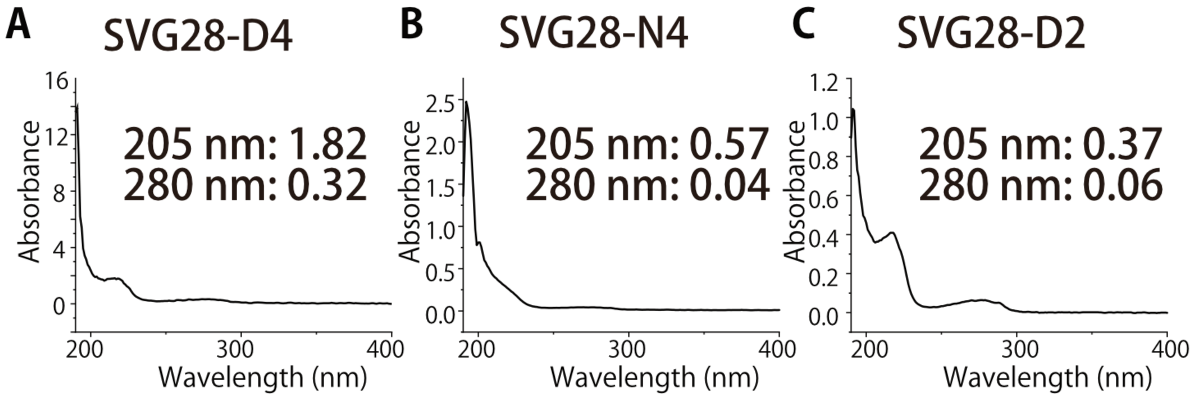

Fig. S6. UV-Vis absorption spectra. A-C. UV-Vis absorption spectra of SVG28-D4 (**A**), SVG28-N4 (**B**), and SVG28-D2 (**C**) expressed using cell-free synthesis. The absorption at 205 nm was used to estimate peptide concentration. Another absorption is at 280 nm is due to the absorption of the Tyr side chain.

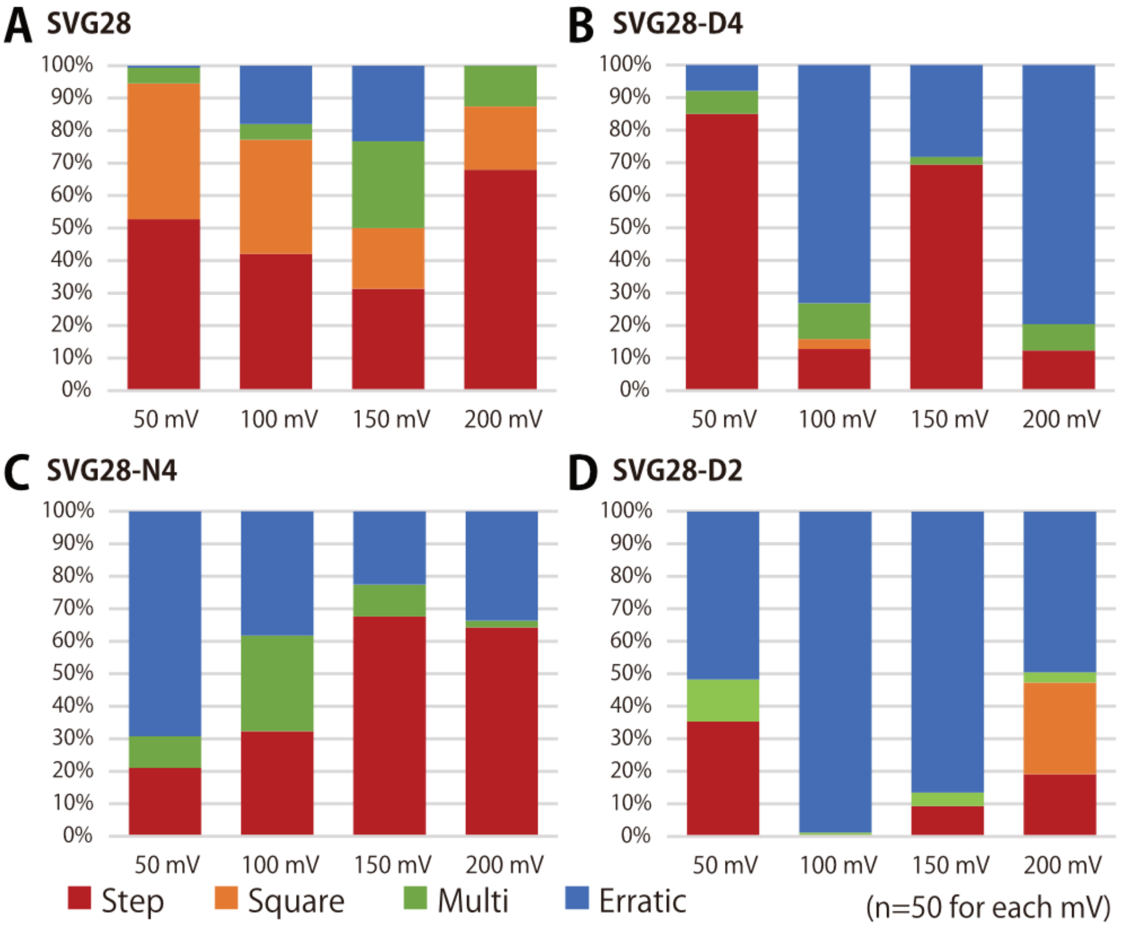

Fig. S7. The duration of each signal type. A-D. The ratio of signal duration of step and square-top (stable pore-formation) and multi and erratic (unstable pore-formation) signals of SVG28 synthesized by solid-phase synthesis (**A**), SVG28-D4 (**B**) SVG28-N4 (**C**), and SVG28-D2 (**D**) under +50, +100, +150, and +200 mV. Although there is variation for each applied voltage, all peptide variants are found to have the ability to open stable pores.

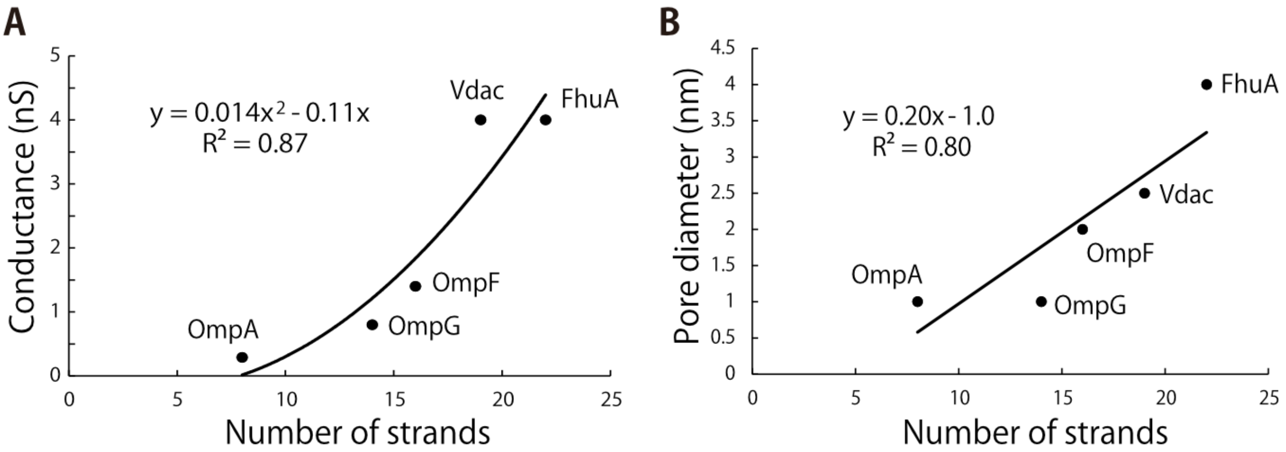

Fig. S8. Estimation of number of monomers and pore diameter. A,B. The relationships in β-barrel membrane proteins CymA, OmpA, OmpF, OmpG, FhuA, and Vdac between the channel conductance and the number of strands (**A**) and the number of monomers and pore diameter (**B**)

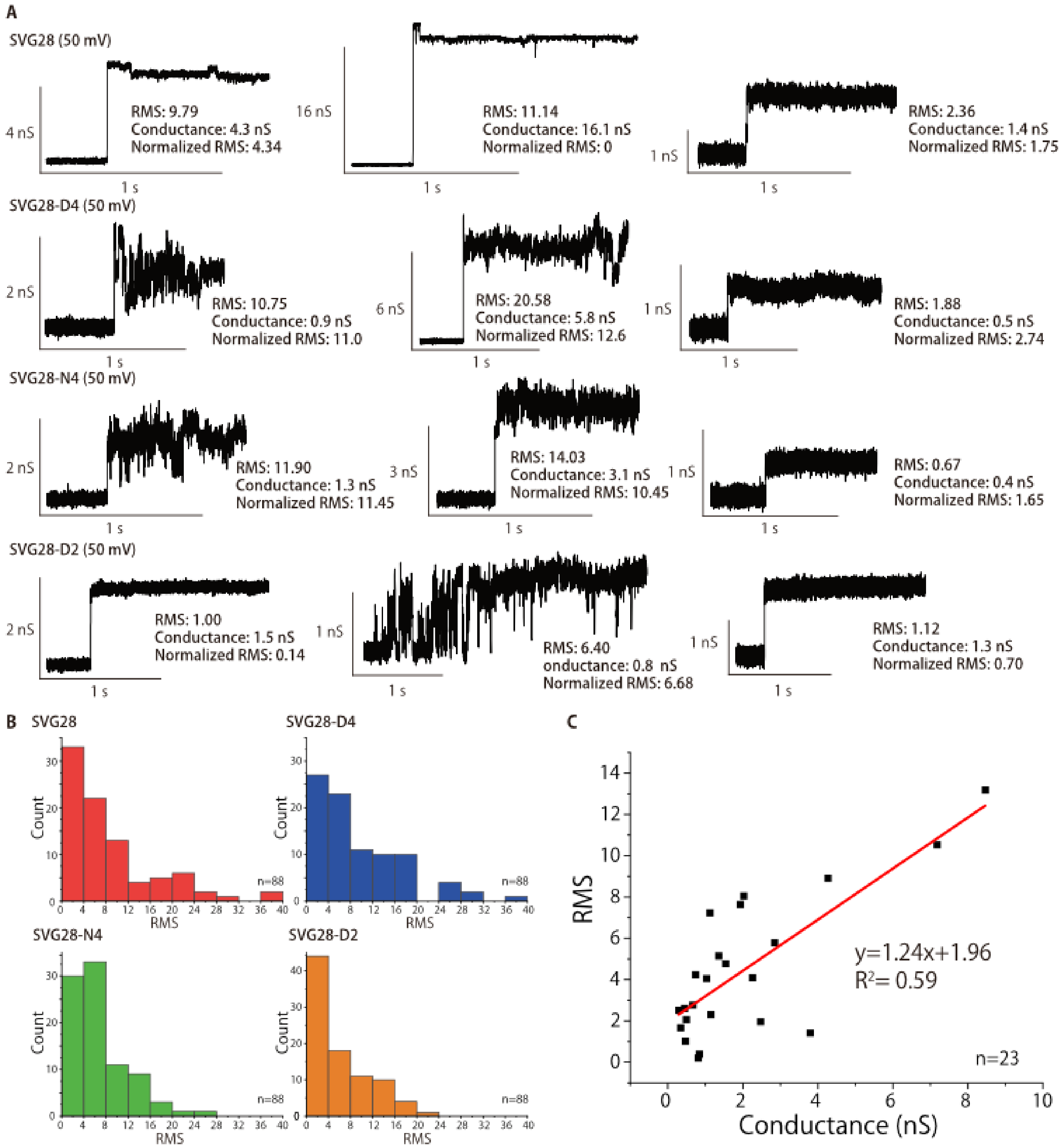

Fig. S9. Noise appears in step signal and RMS evaluations. A. Examples of step signals and noise. The calculated RMS, conductance, and normalized RMS are listed. A comparison of SVG28 (topmost) and SVG28-D4 (second from top) shows that the RMS value is overestimated for larger conductance and vice versa. In addition, the noise-rich signals seen in SVG28-D4 and SVG28-N4 (left and middle) are not suitable for molecular detection because it is difficult to distinguish between signals occurring due to molecular passage and pore-derived noise. **B**. RMS distribution before normalization. SVG28-D4 and N4 have more signals with smaller RMS values and seem to be more suitable for molecular detection. **C**. The diagram of the relationship between current conductance and RMS noise used for the correction (n=23)

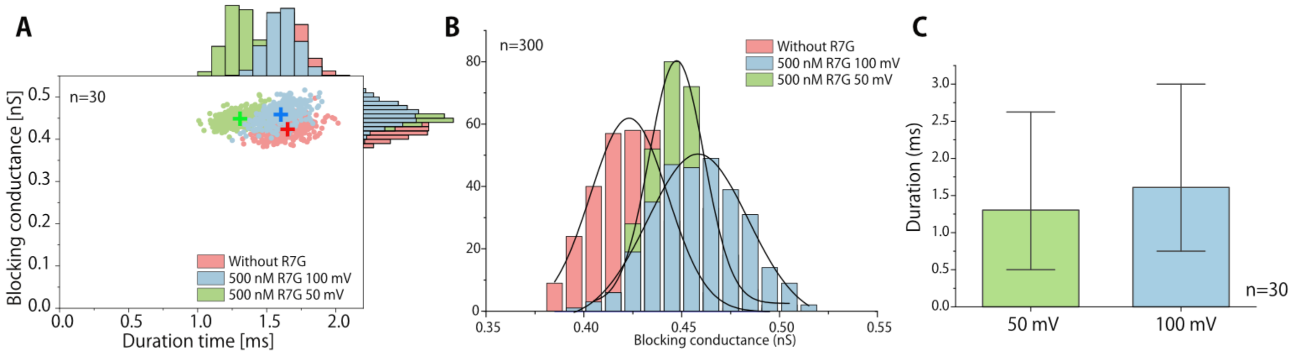

Fig. S10. Details of single-molecule detection. A. Scatter plots of blocking current and duration time after bootstrapping of SVG28-D2 without R7G (control, red, 1.65 ms, 0.42 nS), with R7G under 50 mV (green, 1.30 ms, 0.45 nS), and under 100 mV (blue, 1.60 ms, 0.46 nS) The center of the plot is represented by crosses. The current duration of R7G was increased from 50 mV (1.30 ms) to 100 mV (1.60 ms). No significant difference was observed when the duration time of the translocation signal was compared between 50 mV and 100 mV. **B**. Histogram of blocking current after bootstrapping of SVG28-D2without R7G (control, red, 0.42 nS), with R7G under 50 mV (green, 0.45 nS) and 100 mV (blue, 0.46 nS). **C.** The duration time of the R7G translocation under 50 mV and 100 mV (raw data). The error bars show the 95%confidence interval.

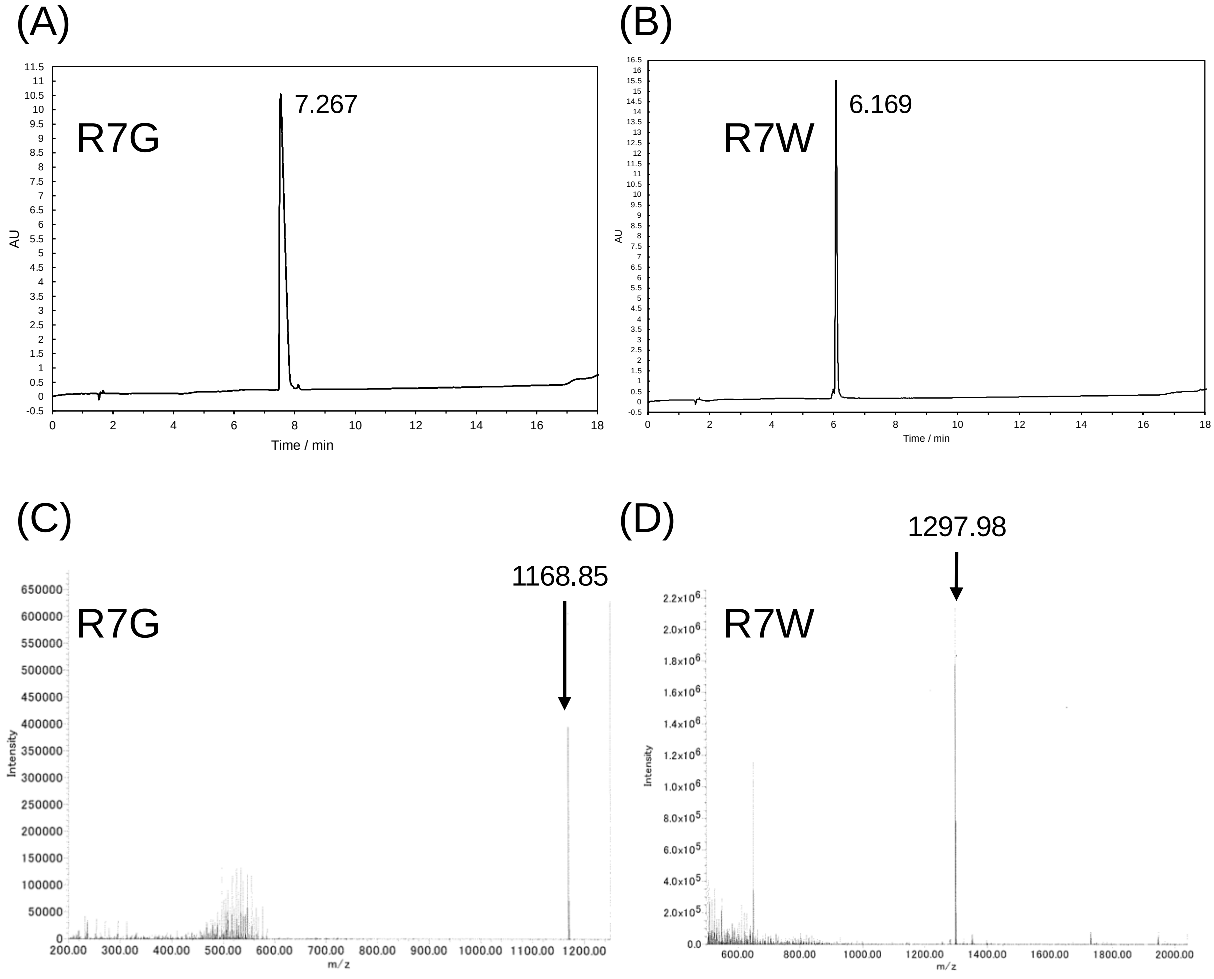

Fig. S11. Solid-phase synthesis of R7X peptide. A,B. HPLC charts of R7G (**A**) and R7W (**B**). For elusion, 0.05% TFA in acetonitrile gradient from 5 to 40% B in 20 min, flow-rate 0.3 ml/min. The retention times of R7G and R7W was 7.267 and 6.169 min, respectively. **C,D.** LC/MS with ESI mode of R7G (**C**) and R7W (**D**). The m/z values were observed at 1168.5 and 1297.8 m/z, respectively (ideal m/z values = 1168.75 [M+H]+ and 1297.81 [M+H]+).

Table. S1. Yield of expressed peptides. The absorption coefficient was calculated for each peptide at 205 nm[43].

Peptide	**Synthesized quantity**
SVG28-D4	1.8 µg/ 20 µL
SVG28-N4	2.4 µg/ 20 µL
SVG28-D2	1.1 µg/ 20 µL

Table. S2. Yield of expressed proteins. These data were provided by website of GeneFrontier (Japan). (https://www.genefrontier.com/en/solutions/purefrex/technology/compare-ver/?noredirect=en_US) The hydrophilic variant of SVG28 (about 3 kDa) is synthesized in the same amount of substance as GFP and DHFR.

Protein	**Synthesized quantity**
DHFR (approx. 18 kDa)	12 µg/ 20 µL
GFP (approx. 27 kDa)	15 µg/ 20 µL
